# Mechanisms of ischemic stroke in patients with intracranial atherosclerosis: A high-resolution magnetic resonance imaging study

**DOI:** 10.3892/etm.2014.1600

**Published:** 2014-03-04

**Authors:** TIANLI GAO, WEI YU, CHUNJIE LIU

**Affiliations:** 1 Department of Neurology, Beijing Anzhen Hospital, Capital Medical University, Beijing 100029, P.R. China; 2 Department of Radiology, Beijing Anzhen Hospital, Capital Medical University, Beijing 100029, P.R. China

**Keywords:** high-resolution magnetic resonance imaging, cerebral artery stenosis, ischemic stroke, intracranial atherosclerosis

## Abstract

High-resolution magnetic resonance imaging (HRMRI) has a unique ability to provide an evaluation of the intracranial artery wall. This study aimed to investigate the possible mechanisms of ischemic stroke in patients with intracranial atherosclerosis using HRMRI. HRMRI was performed on 55 patients (38 male and 17 female) with acute cerebral infarction to investigate the lumen-intruding plaque at the stenotic portion of the middle cerebral artery (MCA) and basilar artery (BA) and to attempt to identify the mechanisms of stroke. Penetrating artery disease (PAD) was diagnosed in 20 patients (36%) and large-artery atherosclerosis (LAA) was diagnosed in 35 patients, including 19 with parent artery plaques occluding a penetrating artery (POPA; 35%) and 16 with artery-to-artery embolisms (29%). Patients with PAD had a higher frequency of hypertension compared with that of the patients with LAA (80 versus 29%; P<0.001), and patients with LAA had a higher frequency of diabetes compared with that of the patients with PAD (40% versus 15%; P=0.054). Magnetic resonance angiography revealed mild to moderate stenosis in the patients with POPA, while border zone infarction and artery-to-artery embolism occurred in the majority of the patients with severe stenosis or occlusion of the MCA and BA. HRMRI has the ability to identify the mechanisms of intracranial atherosclerotic ischemic stroke through the detection of luminal plaques.

## Introduction

Atherosclerosis of the intracranial arteries is frequent and may account for almost one-third of ischemic strokes in the Chinese population (1,2). The underlying mechanisms of cerebral infarction include artery-to-artery embolism, hemodynamic compromise, local branch occlusion or a combination of those conditions (3). Clique histological study of the middle cerebral artery (MCA) has demonstrated that luminal stenosis is frequently caused by ruptured vulnerable plaques, which are characterized by their specific morphology and composition, which comprises a large lipid/necrotic core covered by a thin fibrous cap infiltrated by macrophages and intraplaque hemorrhage (4). Each clique corresponds to a pair of neighboring pixels, and the clique potential is designed to favor similar classes in neighboring pixels. Progression and a greater extent of intracranial atherosclerosis imply a higher risk of recurrence (5).

High-resolution magnetic resonance imaging (HRMRI) has a unique ability to provide information on plaque composition comparable to that obtained by histology (6). Several studies have confirmed the feasibility of using HRMRI to evaluate the intracranial artery wall and have identified the presence of arterial plaques using HRMRI, in cases where magnetic resonance angiography (MRA) showed no lumen abnormality (7–9). In the present study, HRMRI was used to identify the ischemic stroke subtypes of patients with intracranial atherosclerosis and to investigate the possible mechanisms.

## Material and methods

### Study population

A single-center, prospective trial was conducted in the Neurology Department of Beijing Anzhen Hospital, Capital Medical University (Beijing, China) between January 2010 and January 2013. A total of 55 patients with acute cerebral infarction were screened and were subsequently tested for ≥50% MCA and basilar artery (BA) stenosis by cranial MRI and MRA. The ischemic stroke in these patients was presumed to be caused by atherosclerotic disease. Inclusion criteria were as follows: i) unilateral middle cerebral artery stenosis (≥70%) or occlusion due to atherosclerosis were observed while no ipsilateral internal carotid artery stenosis or occlusion could be found; ii) there was infarcted focus within corresponding stenosed artery on MRI. iii) Patients must have at least one of the risk factors for atherosclerosis, including hypertension, diabetes, hyperlipidemia, homocysteine and smoking. Exclusion criteria included: i) patients with ipsilateral internal carotid artery stenosis or occlusion. ii) Non-atherosclerotic cerebral artery stenosis, such as fibromuscular dysplasia, arteritis and dissecting aneurysm. iii) Patients suspected to have symptoms of cardiogenic embolism, including recent myocardial infarction, atrial fibrillation with or without mural thrombus, mitral stenosis or prosthetic valve, dilated cardiomyopathy, sick sinus syndrome, acute bacterial endocarditis and patent foramen ovale. Written informed consent was obtained from all patients. The Ethical Committee of Beijing Anzhen Hospital approved this study (Beijing, China).

### Clinical assessment

All patients underwent a detailed medical history assessment and a physical examination at baseline that included routine blood biochemistry tests, coagulation testing, transthoracic or transesophageal echocardiography, Holter electrocardiography, transcranial Doppler sonography, carotid ultrasound, and computed tomography, MRI and MRA of the brain. Data collected from patients included the following baseline characteristics: Age, gender, vascular risk factors, such as hypertension or history of hypertension or its complications, history of diabetes mellitus (DM) or currently diagnosed DM, hyperlipidemia, history of smoking, previous coronary artery disease and previous cerebrovascular disease.

### Ischemic stroke subclassification

According to the Chinese ischemic stroke subclassification (10), the patients were grouped into two mechanism-based categories: Penetrating artery disease (PAD; no evidence of atherosclerotic plaques or any degree of stenosis in the parent artery) and large-artery atherosclerosis (LAA; plaques in the parent artery occluding a penetrating artery, artery-to-artery embolism or hypoperfusion/impaired emboli clearance).

### MRI protocol and review

All patients were imaged at the Beijing Anzhen Hospital using a Magnetom Verio 3T MRI scanner (Siemens AG, Erlangen, Germany) and an eight-channel brain-array coil. A standardized protocol was used to perform conventional brain T1- and T2-weighted MRI and three-dimensional (3D) time-of-flight (TOF)-MRA. 3D TOF-MRA data were obtained using an axial plane with a repetition time (TR)/echo time (TE) of 21 msec/3.6 msec; flip angle of 18°; field-of-view (FOV) of 220×220 mm; slice thickness of 0.5 mm; and a matrix size of 320×380 pixels. The TOF-MRA scan time was 4 min. MRA data were reconstructed using a dedicated online post-processing tool [multiplanner reconstruction (MPR), maximum intensity (MIP), volume rendering (VR)] to determine the blood vessel architecture.

HRMRI data were acquired from the patients with MCA and BA steno-occlusive lesions along the short axes of the stenotic segments on TOF-MRA images. The lesion site for the evaluation of unilateral MCA stenosis was determined by the interpreting neuroradiologist as the ipsilateral MCA in the symptomatic patients and the side of severe MCA stenosis in the asymptomatic patients. T1- and T2-weighted MRI was centered at the stenosis of the MCA-M1 segment, vertebral artery, BA and their confluences. The MRI parameters were: T1-weighted, double inversion recovery, black blood, two-dimensional turbo spin echo (TSE), TR/TE = 920 msec/27 msec, FOV = 120×120 mm, matrix size = 270×320 pixels, slice thickness = 2.0 mm and 2NEX. For the T2-weighted HRMRI scans, the TSE sequence used a TR/TE of 2,350 msec/78 msec, FOV of 120×120 mm, matrix size of 270×320 pixels, slice thickness of 2.0 mm and 2NEX. The black blood technique with pre-regional saturation pulses of 80- mm thickness to saturate incoming arterial flow was used for the scans. The longitudinal coverage of each artery was 16 mm (eight slices) for the two types of scans, with a scan time of ~3–4 min/scan. The total scan time was ~20 min and the patients remained in the MRI machine for ~30 min.

### Statistical analysis

A χ^2^ test was used to compare frequencies. One-way analysis of variance and Student’s t-test were used for normally distributed variables, whereas the Mann-Whitney U test was used for non-normally distributed variables. All tests of statistical significance were two-sided, with P<0.05 considered to indicate a statistically significant difference. All statistical analyses were performed using SPSS software, version 17.0 (SPSS, Inc., Chicago, IL, USA).

## Results

A total of 55 patients who met the eligibility criteria were recruited to this study. There were 38 males and 17 females with a median age of 58.9 years (standard deviation, ±13.4 years; range, 35–81 years). Among the 55 patients, 17 had vertebrobasilar stenosis (31%) and 38 had MCA stenosis (69%). PAD was diagnosed in 20 of the patients (36%) and LAA was diagnosed in 35 of the patients, which included 19 with a parent artery plaque occluding a penetrating artery (POPA) (35%) and 16 with artery-to-artery embolism and/or hypoperfusion (29%). The baseline characteristics in the two groups were comparable, with the exceptions that the patients with PAD had a higher frequency of hypertension compared with that of the patients with LAA (80 versus 29%; P<0.001) and the patients with LAA had a higher frequency of DM than that of the patients with PAD (40 versus 15%; P=0.054) (Table I). POPA occurred more frequently in the patients with mild to moderate artery stenosis (63%; P<0.05) than in the patients with severe artery stenosis or occlusion (37%). However, the stroke mechanisms of artery-to-artery embolism and/or hypoperfusion were mainly observed in the patients with severe artery stenosis or occlusion (68%) compared with in the patients with mild to moderate artery stenosis (13.3%; P=0.060).

**Figure 1:**
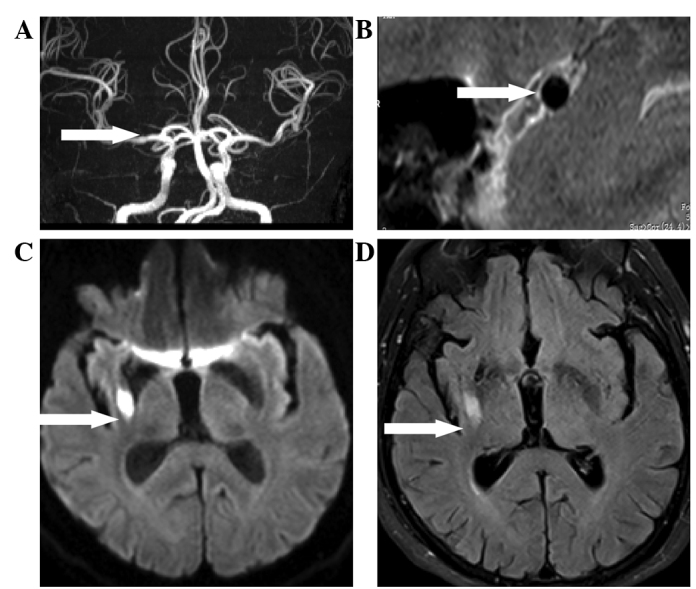
A 72-year-old male presented with left hemiparesis and dysarthria. (A) Right MCA wall MRA indicates mild-grade MCA stenosis. (B) T2-weighted HRMRI cross-sectional image of the MCA (M1 segment) reveals a normal circular lumen with no wall thickening. (C) A single small infarction of a penetrating artery is shown using DWI. (D) T2 fluid-attenuated inversion-recovery imaging indicates an infarction lesion located at the same position, corresponding to the territory of a penetrating artery of the right MCA. The arrows represent the position of the lesion. MCA, middle cerebral artery; HRMRI, high-resolution magnetic resonance imaging; DWI, diffusion-weighted imaging.

**Figure 2:**
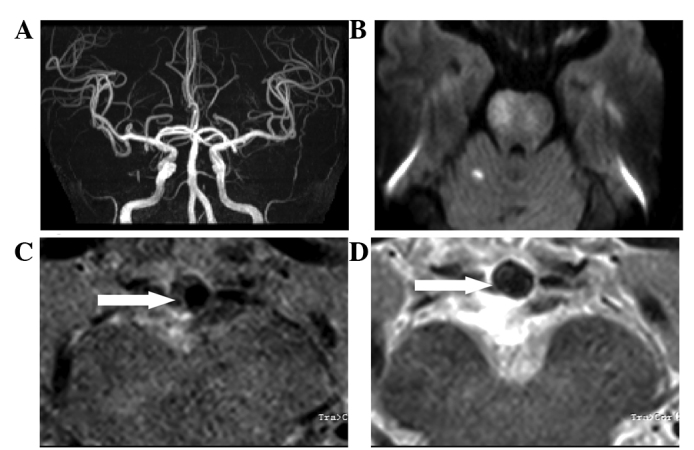
A 60-year-old female presented with an episode of sudden onset left face, arm and leg weakness. (A) MRA revealed an irregular mild BA stenosis close to the pontine paramedian artery. (B) An acute high-intensity infarction was demonstrated on the axial DWI image at the level of the right lateral pontine. (C and D) Cross-sectional T1- and T2-weighted HRMRI at the level of the BA revealed a lipid-rich necrotic eccentric plaque with low or iso-signal density. The arrows represent the position of the lesion. MRA, magnetic resonance angiography; BA, basilar artery; DWI, diffusion-weighted imaging; HRMRI, high-resolution magnetic resonance imaging.

**Figure 3:**
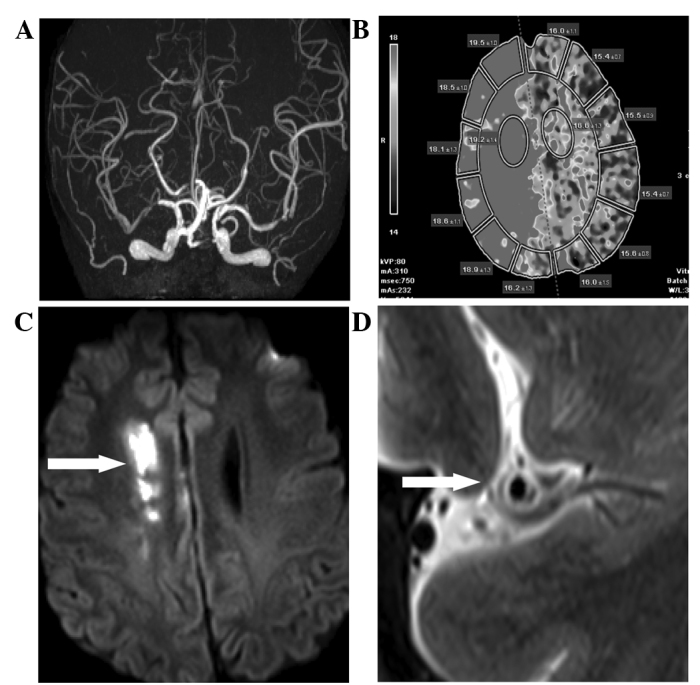
A 71-year-old female patient presented with dysarthria and mild left hemiparesis. (A) MRA from the right proximal MCA (M1 segment) showed a focal severe stenosis. (B) DWI confirmed internal border-zone infarction, which corresponded to the right MCA territory with the underlying mechanism of hypoperfusion and impaired emboli clearance. (C) Computed tomography perfusion imaging also demonstrated relatively low cerebral blood flow, and elevated time-to-peak in a region of the right MCA indicated the potential of hypoperfusion. (D) The T2-weighted HRMRI cross-sectional lumen image showed a hyposignal eccentric core surrounded by a high-signal thickened wall. The arrows represent the position of the lesion. MRA, magnetic resonance angiography; MCA, middle cerebral artery; DWI, diffusion-weighted imaging; HRMRI; high-resolution magnetic resonance imaging.

HRMRI enabled the detection of the lumen wall, and the MCA and BA were clearly observed in all cases. The cross-sectional imaging findings of patients who were diagnosed with LAA indicated that the presence of focal arterial wall thickening was consistent with a plaque on the level of the MCA and BA stenotic area on MRA images. The plaque appeared as a crescent-shaped or eccentric thickening surrounding a circular lumen. As shown in Fig. 1, in a 72-year-old male patient, MRA revealed relatively mild stenosis in the MCA-M1 segment, while HRMRI showed no lumen abnormality. A high-intensity infarct lesion revealed by diffusion-weighted imaging (DWI) was present in the lateral striate arterial territory, indicating that this was a case of the PAD stroke subtype. HRMRI findings of the lumen elucidated the underlying mechanisms in which lipohyalinosis or microatheroma may be the etiology for PAD. Furthermore, in a 60-year-old female patient, MRA showed relatively mild to moderate stenosis in the BA where an eccentric plaque located in the lumen wall was shown to occlude the paramedian pontine arteries by HRMRI. An acute ischemic infarction was verified by DWI (Fig. 2). This was indicated to be a typical case, where the infarction was confined to the territory of a single branch artery or a few penetrating branches that were occluded by the plaques of their parent arteries. Borderzone infarction and artery-to-artery embolism occurred in the most severe stenosis and occlusion of the MCA and BA. Computed tomography perfusion imaging of a 71-year-old female patient demonstrated relatively low cerebral blood flow and elevated time-to-peak in a region of the MCA, indicating the potential mechanism of hypoperfusion (Fig. 3).

## Discussion

Intracranial atherosclerotic disease causes ischemic strokes, and the rates of recurrent vascular ischemic events and vascular mortalities are very high (11). However, intracranial atherosclerosis, which affects cerebral arteries such as the BA and MCA, remains an infradiagnosed and understudied disease (12). The diagnosis of intracranial stenosis is traditionally dependent on conventional angiography and several reliable noninvasive diagnostic methods, including transcranial color-coded duplex sonography, MRA and computed tomography angiography (13,14). Cross-sectional HRMRI is a promising technique for imaging carotid plaques, with a sensitivity of 85% and a specificity of 92% for identifying soft plaques (necrotic core or hemorrhage) (15–17). HRMRI has consistently emerged as a potential technique for imaging atherosclerotic plaques in the intracranial arteries (7). This imaging technique may provide information about the histopathological nature of the intracranial atherosclerotic lesion responsible for arterial narrowing (9).

As no underlying cause is found in >30% of stroke cases, HRMRI detection of intracranial atherosclerotic lesions may have significant clinical implications (3). Detection of the vessel wall of arteries such as the MCA and BA may improve the ability to identify advanced but unrecognized intracranial atherosclerotic disease (18). In the present study, the features of the MCA and BA luminal wall in patients who had suffered cerebral infarction were explored. Recognizing the characteristics of LAA and PAD may contribute to improved risk stratification and allow aggressive interventions to be targeted at patients with plaques that are prone to rupture (19–21). A previous study of carotid artery plaques has demonstrated a significant correlation between plaque characteristics identified by HRMRI and subsequent stroke patterns (19). In the present study, HRMRI clearly confirmed the presence of a reduced arterial lumen associated with a focal wall thickening and plaques at the level of MCA and BA stenosis, suggesting that the HRMRI technique was useful in determining the etiology of PAD and POPA.

In a comparison of the patients with PAD and those with LAA, it was observed that patients with PAD had a higher frequency of hypertension whereas patients with LAA had a higher frequency of DM. Aging and chronic hypertension are risk factors for large-artery atherosclerosis (22). Such changes include replacement of the smooth muscle cells by fibro-hyaline material with thickening of the wall and narrowing of the vascular lumen. Arteriolosclerosis may be one of the reasons that the blood supply to the white matter is altered, and this vascular change may lead to localized ischemic areas of necrosis and cavitations (14). Previous studies have shown a high frequency of intracranial stenosis in diabetic Caucasian patients, an independent association of type II DM to a greater extent of intracranial LAA, and a significantly higher number of diseased vessels in diabetic patients compared with that in nondiabetic patients (13,23). The association between diabetes and more diffuse and advanced intracranial atherosclerosis is unclear. Consequently, among the traditional vascular risk factors, diabetes appears to play a preeminent role in intracranial macroangiopathy in the Chinese population (24). Additionally, the presence of intracranial LAA disease contributes to a poorer outcome for patients with LAA disease, which may be stratified as very high risk in secondary prevention (25).

In conclusion, stroke patterns of intracranial atherosclerotic arteries are complicated and mainly include LAA and PAD. HRMRI has the ability to identify the mechanisms behind intracranial atherosclerotic ischemic stroke by showing the luminal wall. It may also provide a useful tool in risk stratification and the selection of candidates for invasive therapies.

## Figures and Tables

**Table I:** Baseline characteristics of patients with PAD and LAA.

Characteristic	PAD (n=20)	LAA (n=35)	P-value
Mean age (years; SD, range)	62 (14.5, 35–81)	58 (12.1, 47–75)	0.569
Gender (male/female)	13/7	25/10	0.619
Hypertension	16 (80%)	10 (29%)	<0.001
DM	3 (15%)	14 (40%)	0.054
Hyperlipidemia	4 (20%)	6 (17%)	0.792
History of smoking	8 (40%)	13 (37%)	0.834
History of CAD	5 (25%)	9 (26%)	0.953
History of CVD	3 (15%)	7 (20%)	0.644

PAD, penetrating artery disease; LAA, large-artery atherosclerosis; DM, diabetes mellitus; CAD, coronary artery disease; CVD, cerebrovascular disease.
